# Sphingomyelin synthase 2 promotes an aggressive breast cancer phenotype by disrupting the homoeostasis of ceramide and sphingomyelin

**DOI:** 10.1038/s41419-019-1303-0

**Published:** 2019-02-15

**Authors:** Kehong Zheng, Zetao Chen, Haizhan Feng, Ying Chen, Cheng Zhang, Jinlong Yu, Yunfeng Luo, Liang Zhao, Xiancheng Jiang, Fujun Shi

**Affiliations:** 1 Department of General Surgery, Zhujiang Hospital, Southern Medical University, Guangzhou, China; 2 Division of Laboratory Medicine, Zhujiang Hospital, Southern Medical University, Guangzhou, China; 3 Department of Pathology, Nanfang Hospital, Southern Medical University, Guangzhou, China; 4 Department of Pathology, School of Basic Medical Sciences, Southern Medical University, Guangzhou, China; 5 Department of Cell biology, Downstate Medical Centre, State University of New York, New York, NY USA

## Abstract

Breast cancer is the most common type of carcinoma in women worldwide, but the mechanisms underlying tumour development and progression remain unclear. Sphingomyelin synthase 2 (SGMS2) is a crucial regulator involved in ceramide (Cer) and sphingomyelin (SM) homoeostasis that is mostly studied for its role in lipid metabolism. Our primary study indicated that high SGMS2 expression is associated with breast cancer metastasis. Gain- and loss-of-function assays in vitro and in vivo revealed that SGMS2 promotes cancer cell proliferation by suppressing apoptosis through a Cer-associated pathway and promotes cancer cell invasiveness by enhancing epithelial-to-mesenchymal transition (EMT) initiation through the TGF-β/Smad signalling pathway. Further study determined that SGMS2 activated the TGF-β/Smad signalling pathway primarily by increasing TGF-β1 secretion, which was likely associated with aberrant expression of SM. Thus, our findings indicate that SGMS2-mediated activation of the TGF-β/Smad signalling pathway is important in breast cancer progression, which provides new insight into the mechanisms underlying breast cancer metastasis and suggests a possible anticancer therapy for breast cancer.

## Background

Breast cancer is the most common malignancy and one of the leading causes of cancer-related death and reduced disability-adjusted life years for women^1^. Although numerous studies have determined that tumour metastasis is the most important reason for the death of patients with breast cancer, the mechanism underlying tumour metastasis is still not clear^2,3^. Thus, improving our understanding of the molecular mechanisms underlying breast cancer progression may help us develop effective methods to manage this disease.

Sphingomyelin synthase (SGMS) is a transferase that regulates the synthesis of sphingomyelin (SM) from ceramide (Cer)^4^. Although SGMS has three homologues, namely, SGMS1, SGMS2 and SGMS-related protein (SGMSr), only SGMS1 and SGMS2 promote SM synthesis, while SGMSr promotes synthesis of the SM analogue ceramide phosphoethanolamine^5^. Cer plays a vital role in regulation of cell apoptosis^6^. A previous study determined that upregulating SGMS2 significantly decreased the expression of Cer, which led to aberrant cell apoptosis activity, consequently promoting cell proliferation^7^. It is well-known that SM is the major component of various biological membranes; it participates in regulation of membrane stability and cell secretion activity. Studies in many types of cancer have determined that SM promotes cancer development and progression by regulating cell proliferation and migration potential^5^. Thus, we suppose that SGMS2 is quite important in promotion of an aggressive breast cancer cell type by regulating the expression of Cer and SM. However, the mechanism by which SGMS2 promotes breast cancer development and progression remains unknown.

Due to the heterogeneity of breast cancer, we generally characterise several intrinsic molecular breast cancer subtypes according to the tumour gene-expression profile, such as luminal, basal-like, normal-like and triple-negative breast cancer^8^. Prognosis and treatment differ between molecular subtypes^9^. Given this context, two distinct human breast cancer cell lineages were used in our research: non-invasive breast cancer cells (MCF-7) corresponding to the epithelial subtype and invasive breast cancer cells (MDA-MB-231) corresponding to the mesenchymal subtype^10^. We investigated the role of SGMS2 in proliferation and migration of breast cancer cells through both in vitro and in vivo studies and analysed the related signalling pathways that enhance the aggressive of breast cancer cells.

## Materials and methods

### Breast cancer cell lines and tumour tissue samples

The breast cancer cell lines MCF-7 and MDA-MB-231were obtained from the Cell Bank of the Chinese Academy of Science (Shanghai, China) and maintained as the protocol required. All cells were authenticated by short-tandem repeat profiling after receipt and were propagated for less than 6 months after resuscitation. The cells were grown in RPMI 1640 medium (Life Technologies Corporation; Grand Island, NY) supplemented with 10% foetal bovine serum (Life Technologies Corporation; Grand Island, NY).

Fresh primary breast cancer specimens and paired noncancerous breast tissue specimens were provided by the Department of General Surgery, Zhujiang Hospital of Southern Medical University in Guangzhou, China. Each patient was diagnosed with primary invasive ductal carcinoma of the breast and received modified radical mastectomy in Zhujiang Hospital between Jan 2016 and March 2017. The pathological diagnosis was made by the Department of Pathology of Zhujiang Hospital. The study was approved by the Ethics Committee of Southern Medical University, and all aspects of the study complied with the criteria of the Declaration of Helsinki. The Committee approved the collection of tissue without requiring informed consent, given that the data would be analysed anonymously.

### RNA isolation, reverse transcription and quantitative real-time PCR

Total RNA was extracted using Trizol (Invitrogen; Carlsbad, CA). To quantify the expression of SGMS2, the total RNA was subjected to polyadenylation and reverse transcription (RT) using a ThermoScript^TM^ RT-PCR System (Invitrogen). Real-time PCR analysis was carried out using SYBR Green PCR master mix (Applied Biosystems; Foster City, CA) on an ABI 7500HT system. GAPDH (for cell samples) and RPLP0 (for tumour tissue samples) snRNA were used as endogenous controls. All samples were normalised to internal controls, and fold changes were calculated through relative quantification (2^−^^ΔΔCT^). The primers used are shown in Supplementary Table S1.

### Western blot analysis

Protein expression was assessed by immunoblot analysis of cell and tissue lysates (20–60 μg) in RIPA buffer in the presence of rabbit antibodies against SGMS2, E-cadherin, N-cadherin, β-catenin, vimentin, Snail, TGF-β1, Smad2 and phosphorylated-Smad2 (p-Smad2) and mouse antibodies against fibronectin and GAPDH (1:1000; Cell Signalling Technology; Danvers, MA).

### Immunofluorescence assay

Cells were cultured on coverslips overnight, fixed with 4% paraformaldehyde for 30 min, and then treated with 5% Triton X-100 (Sigma, USA) for 15 min. After being blocked in 10% normal blocking serum (Sigma, USA) at room temperature for 15 min, the slides were incubated with rabbit antibody against E-cadherin (1:150) (Cell Signalling Technology; USA) and rabbit antibody against Vimentin (1:100) (Proteintech, USA) at 4 °C overnight, followed by three washes with phosphate-buffered saline. Cover slips were incubated with a fluorescein isothiocyanate-conjugated anti-rabbit or anti-mouse antibody or a Texas Red-conjugated anti-mouse or anti-rabbit antibody (1:200) (SantaCruz Biotech; USA) for 30 min at room temperature and then stained with 6-diamidino-2-phenylindole (DAPI, Invitrogen).

### Enzyme-linked immunosorbent assay

Cytoplasm and supernatants from MDA-MB-231 and MCF-7 cells were collected, and the cell number was determined. The total Cer and SM levels in the cytoplasm were measured using a human ceramide and SM ELISA Kit (Enzyme-linked Biotechnology, Shanghai, China) according to the manufacturer’s instructions. The total level of secretory TGF-β1 in the supernatant was measured using a human TGF-β1 ELISA Kit (Enzyme-linked Biotechnology, Shanghai, China) according to the manufacturer’s protocol. The cytokine expression level (pg/ml) per 10^5^ cells was analysed.

### Tumour growth assay

Four-to-five-week-old male BALB/c nude mice were purchased from the Laboratory Animal Services Centre at the Southern Medical University. Animal handling and experimental procedures were approved by the Animal Experimental Ethics Committee of Southern Medical University. For the tumour growth assay, 1 × 10^7^ SGMS2 overexpression cells were injected subcutaneously into the right back of nude mice, while the control cells were correspondingly injected into the left back (*n* = 5/group). To facilitate oestrogen-dependent tumour establishment, each mouse in the MCF7 cell groups received 17-estradiol (0.72 mg/ pellet, 60 day release; Innovative Research of America). Tumour volume was calculated using the following formula: *V* = 0.5 × *D* × *L*^2^, where *V* represents volume, *D* represents the longitudinal diameter and *L* represents the latitudinal diameter.

### Statistical analysis

Data were analysed using SPSS version 19.0 software (SPSS, Chicago, IL). Nonparametric test (Wilcoxon and Mann–Whitney) was used to analyse clinical data. Student’s *t* test and one-way ANOVA were carried out for comparisons between two groups. Paired-samples *t* test was used to analyse paired data. All statistical tests were two-sided, and statistical significance was established at *P* < 0.05.

For additional Materials and methods please refer to the Supplementary materials.

## Results

### SGMS2 is increased in breast cancer metastasis

**Fig. 1:**
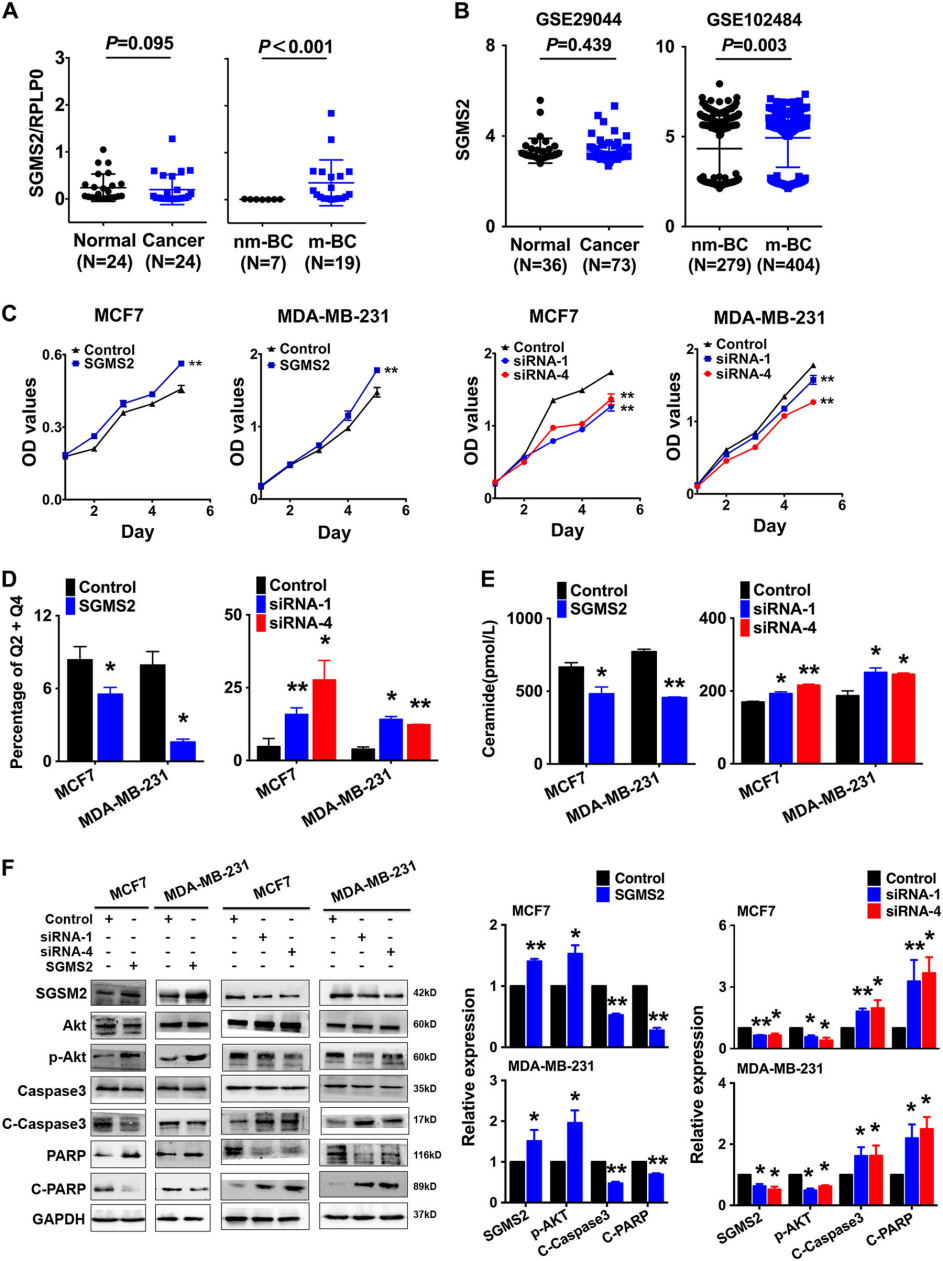
SGMS2 promotes the proliferation of breast cancer cells by disrupting the Cer-associated apoptosis pathway. **a** Real-time PCR analysis of SGMS2 in 30 human breast cancer tissue specimens. SGMS2 expression was normalised to RPLP0. “nm-BC” denotes breast cancer tissue from patients without metastasis. “m-BC” denotes breast cancer tissue from patients with lymph node metastasis. **b** The expression of SGMS2 in Gene Expression Omnibus (GEO) GSE102484 (right) and GSE29044 (left) datasets. **c** The effect of SGMS2 on cell proliferation was evaluated by CCK-8 analysis. **d** Annexin V staining showing the percentage of cells undergoing apoptosis in treated MCF7 and MDA-MB-231 cells, as indicated. **e** ELISA assay of Cer expression in cells treated with an SGMS2 ORF construct, siRNA and their controls. **f** Western blot analysis was performed to detect the expression of Cer-associated apoptosis pathway proteins. Representative figures are shown in the left panel. Bars in the right panel represent the expression of genes normalised to the reference gene GAPDH or Akt, Caspase3 and PARP expression and control groups. “C-” denotes “Cleavage-”. The results are from three independent experiments. * *P* < 0.05 and ** *P* < 0.01 vs. Control

SGMS2 expression was significantly higher in breast cancer tissues from patients with lymph node or distant metastasis compared with non-metastasis samples (*P* < 0.001, Fig. 1a, right). However, there was no significant difference in SGMS2 expression between breast cancer and adjacent normal tissue (*P* *=* 0.095, Fig. 1a left). The expression of SGMS2 was also investigated in Gene Expression Omnibus (GEO) GSE102484 (*P* = 0.003, Fig. 1b right) and GSE29044 (*P* = 0.439, Fig. 1b left) datasets and the results were consistent with the above-described results. These findings suggest that the alteration of SGMS2 in breast cancer is associated with tumour metastasis but not tumorigenesis.

### SGMS2 promotes the proliferation potential of breast cancer cells in vitro by disrupting the Cer-associated apoptosis pathway

Further studies were applied to determine the effects of SGMS2 on the bioactivities of breast cancer cells. Real-time PCR (Fig. S1) and immunoblotting (Fig. 1f) assays were used to confirm the efficiency of *SGMS2* ORF constructs and anti-*SGMS2* small interfering RNA oligonucleotides (siRNA) in MCF-7 and MDA-MB-231 cells. CCK-8 assays revealed that SGMS2 significantly promoted the proliferation of both MCF-7 and MDA-MB-231 cells (*P* < 0.05, Fig. 1c). In addition, annexin V staining showed that SGMS2 decreased the percentage of cells undergoing apoptosis in both cell lines, whereas anti-SGMS2 promoted cell apoptosis (*P* < 0.05, Fig. 1d, Supplementary Fig. S2).

It is well-known that SGMS2 is a key enzyme in the hemeostasis of Cer, which is tightly associated with cell apoptosis^5,11^. Many studies have revealed that ceramide plays a pivotal role in cell apoptosis via the P-AKT → caspase-3 → PARP signalling pathway in several types of diseases, including renal cell carcinoma^12^, hepatocellular carcinoma^13,14^ and retinopathy^15^. To determine the mechanism underlying the SGMS2-induced increase in cell proliferation, we investigated the expression of Cer and activation of the Cer-associated apoptosis pathway. Enzyme-linked immunosorbent assays demonstrated that SGMS2 significantly decreased the expression of Cer, whereas suppression of SGMS2 presented the opposite result (Fig. 1e). Immunoblot assays showed that overexpression of SGMS2 promoted Akt phosphorylation and decreased the cleavage of Caspase3 and PARP. By contrast, suppression of SGMS2 inhibited Akt phosphorylation but promoted the cleavage of Caspase3 and PARP (Fig. 1f).

### SGMS2 promotes migration/invasion of breast cancer via EMT triggered by the TGF-β/Smad signalling pathway

**Fig. 2:**
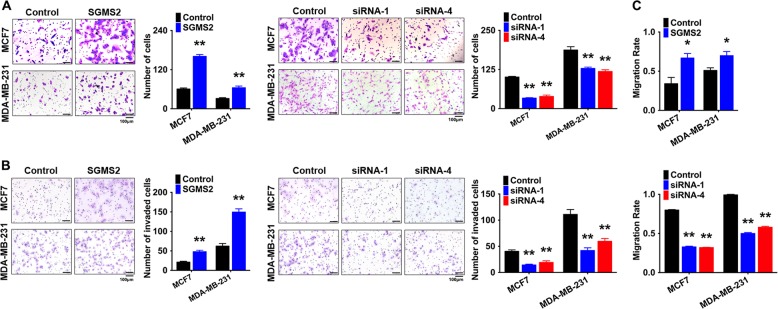
Transient SMGS2 expression promotes the migration and invasiveness of breast cancer cells in vitro. **a** Transwell assay of cells treated with an SGMS2 ORF construct, siRNA and their controls. Representative figures are shown in the left panel, and bars in the right panel represent the number of invaded cells; the cells in five randomly selected fields were counted under a microscope. **b** Tumour invasion assay of cells treated with an SGMS2 ORF construct, siRNA and their controls. Representative figures are shown in the left panel, and bars in the right panel represent the number of invaded cells; the cells in five randomly selected fields were counted under a microscope. **c** Wound-healing assay of the treated cells, as indicated. Bars represent the migration rates of treated cells. The distances travelled by treated cells were measured relative to that travelled by control cells. *P < 0.05 and **P < 0.01 vs. Control

**Fig. 3:**
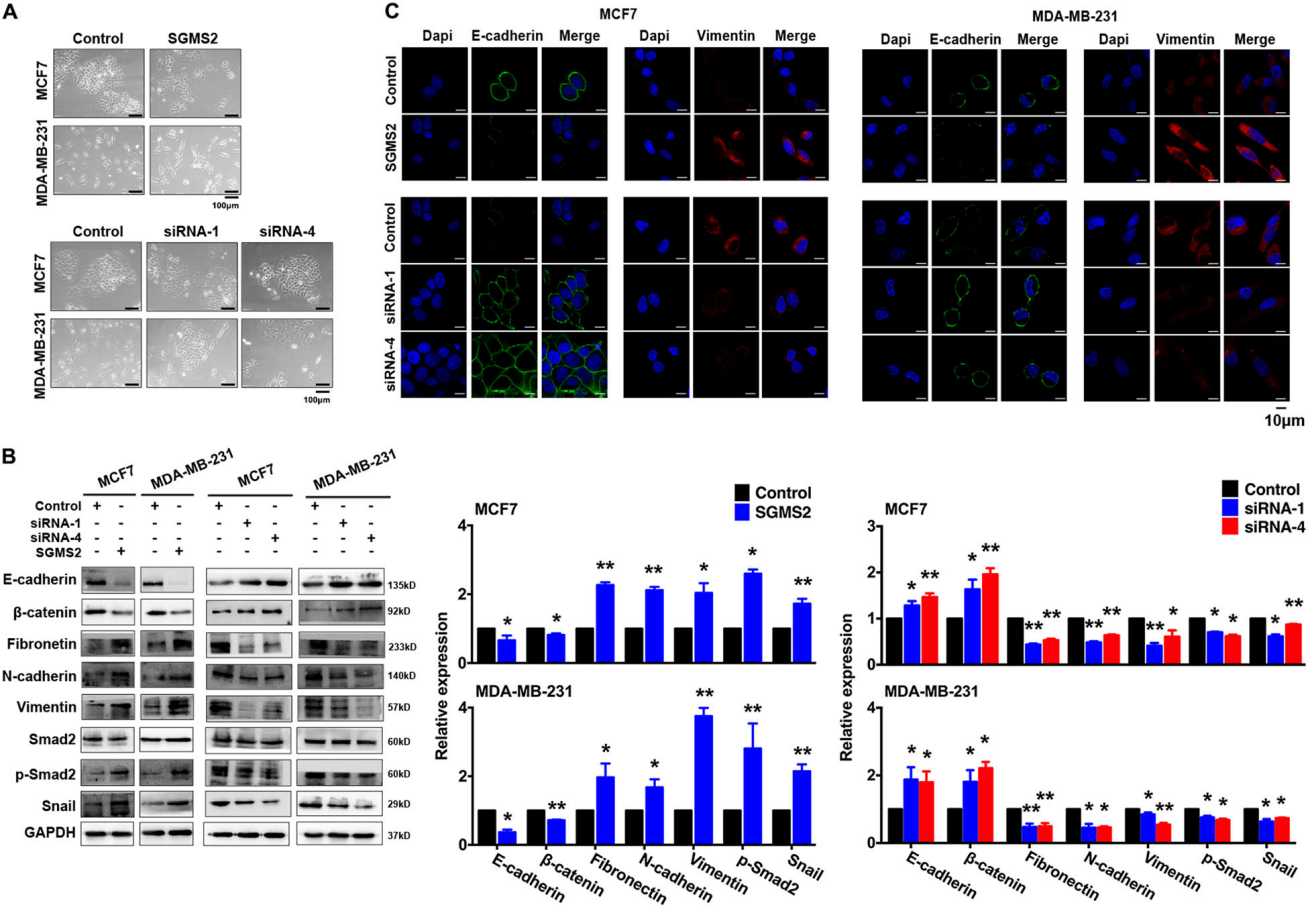
SGMS2 promotes EMT in breast cancer cells by activating the TGF-β/Smad signalling pathway. **a** Cell morphology assay of breast cancer cells treated with SGMS2 ORF construct, siRNA and their controls. Representative figures are shown. **b** Western blot analysis was performed to detect EMT and TGF-β/Smad signalling pathway-associated proteins. Representative figures are shown. Bars in the right panel represent the expression of genes normalised to reference gene GAPDH or Smad2 expression and control groups. The results are from three independent experiments. **c** Immunofluorescence assay of E-cadherin and Vimentin expression in treated cells, as indicated. Representative figures are shown. Scale bars represent 10 μm. ^*^P < 0.05 and ^**^P < 0.01 vs. Control

Transwell and tumour invasion assays revealed that SGMS2 significantly enhanced the migration and invasion potential of both MCF-7 and MDA-MB-231 cells (*P* < 0.05, Fig. 2a, b). Wound-healing assays showed that SGMS2 also promoted the motility potential of MCF-7 and MDA-MB-231 cells (*P* < 0.05, Fig. 2c, and Supplementary Fig. S3). Cell morphology assays showed that only MCF-7 cells significantly transformed into a more mesenchymal phenotype after transfection with *SGMS2* ORF constructs. By contrast, inhibiting *SGMS2* expression only transformed MDA-MB-231 cells into a more epithelial phenotype (Fig. 3a). However, further immunoblotting assays determined that SGMS2 inhibited the expression of epithelial markers (E-cadherin and β-catenin) and stimulated the expression of mesenchymal markers (Fibronectin, N-cadherin and Vimentin) in both cell lines. By contrast, inhibition of SGMS2 expression stimulated the expression of epithelial markers but suppressed the expression of mesenchymal markers. (Fig. 3b). Additional immunofluorescence assays determined that SGMS2 could facilitate epithelial-to-mesenchymal (EMT) transition in both of the breast cancer cell lines (Fig. 3c). It has been well-characterised that EMT is tightly involved in activation of the TGF/Smad signalling pathway. To determine the mechanism underlying the role of SGMS2 in promoting EMT in breast cancer cells, we investigated the TGF-β/Smad signalling pathway. Immunoblotting assay findings suggested that SGMS2 promoted Smad2 phosphorylation, whereas silencing of SGMS2 reduced the expression of phosphorylated Smad2 (Fig. 3b). In addition, SGMS2 overexpression promoted the expression of Snail, a key trigger of epithelial-to-mesenchymal transition mediated by TGF-β/Smad signalling, while suppression of SGMS2 decreased Snail expression (Fig. 3b).

### SGMS2 exerts tumour-promoting effects primarily by enhancing TGF-β1 secretion and activating TGF-β/Smad signalling pathway

**Fig. 4:**
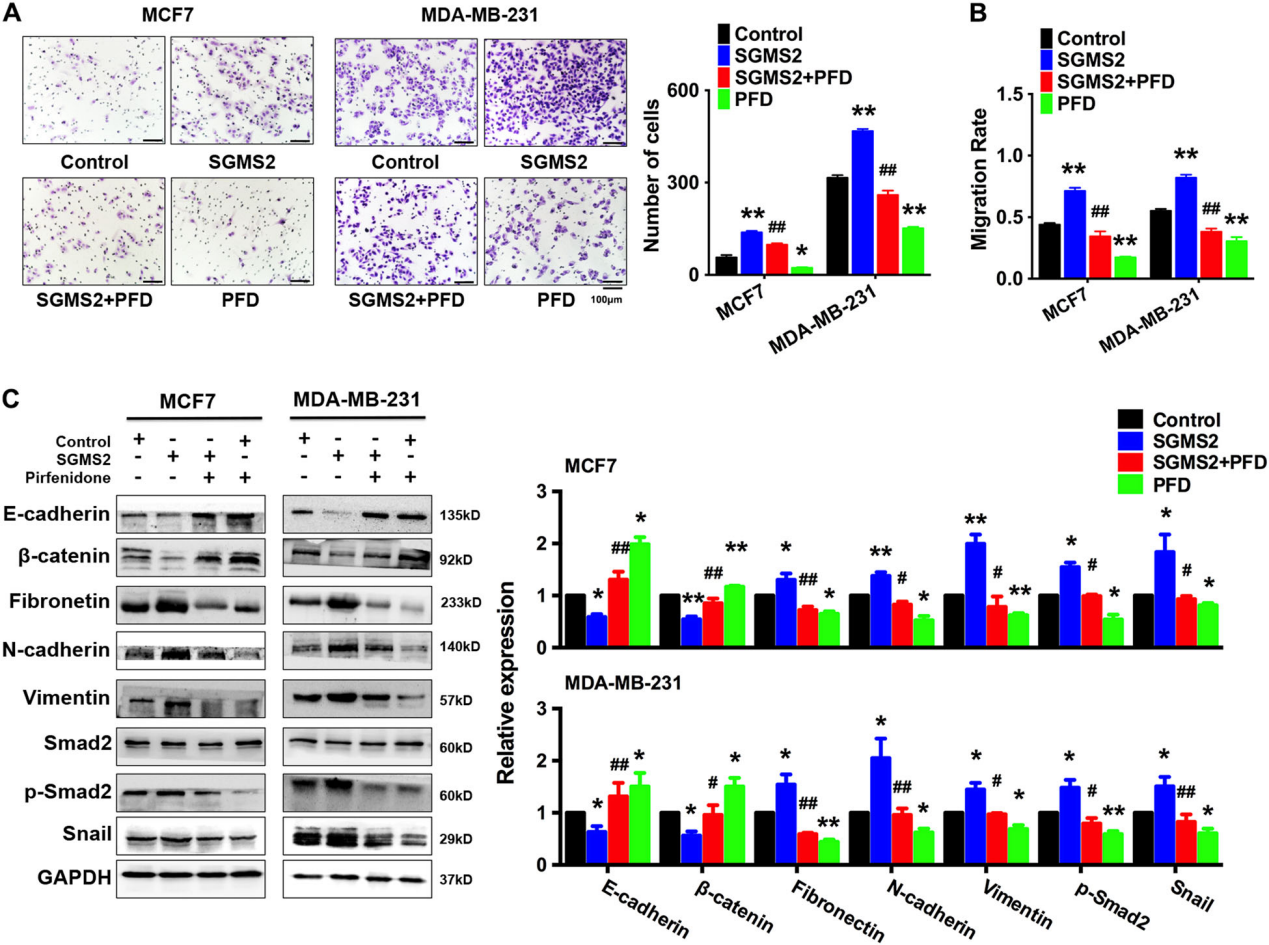
TGF-β/Smad signalling pathway plays a crucial role in SGMS2-mediated promotion of cell migration. **a** Transwell assay of cells with SGMS2 overexpression and/or treated with pirfenidone. Representative figures are shown in the left panel; Bars in the lower panel represent the number of invasive cells. PFD indicates pirfenidone. The cells were counted under a microscope in five randomly selected fields. **b** Wound-healing assay of cells with SGMS2 overexpression treated with pirfenidone. The distance travelled by treated cells was measured relative to that travelled by control cells. **c** Western blot analysis was performed to detect EMT and TGF-β/Smad signalling pathway-associated proteins. Representative figures are shown in the left panel. Bars in the right panel represent the expression of genes normalised to reference gene GAPDH or Smad2 expression and control groups. PFD indicates pirfenidone. *P < 0.05 and **P < 0.01 vs. control; ^#^*P* < 0.05 and ^##^*P* < 0.01 vs. SGMS2

To determine the role of the TGF-β/Smad signalling pathway in the tumour-promoting effects of SGMS2, we specifically disrupted the TGF-β/Smad signalling pathway using pirfenidone in SGMS2 transient overexpression breast cancer cells. We found that blocking the TGF-β/Smad signalling pathway removed the SGMS2-mediated promotion of motility and the migration potential of MCF-7 and MDA-MB-231 cells (Fig. 4a, b, and Supplementary Fig. S4). Immunoblotting assays showed that blocking the TGF-β/Smad signalling pathway reversed the SGMS2-mediated alteration in EMT signature (Fig. 4c). These findings strongly suggest that the TGF-β/Smad signalling pathway plays a vital role in the SGMS2-induced invasiveness of breast cancer cells.

**Fig. 5:**
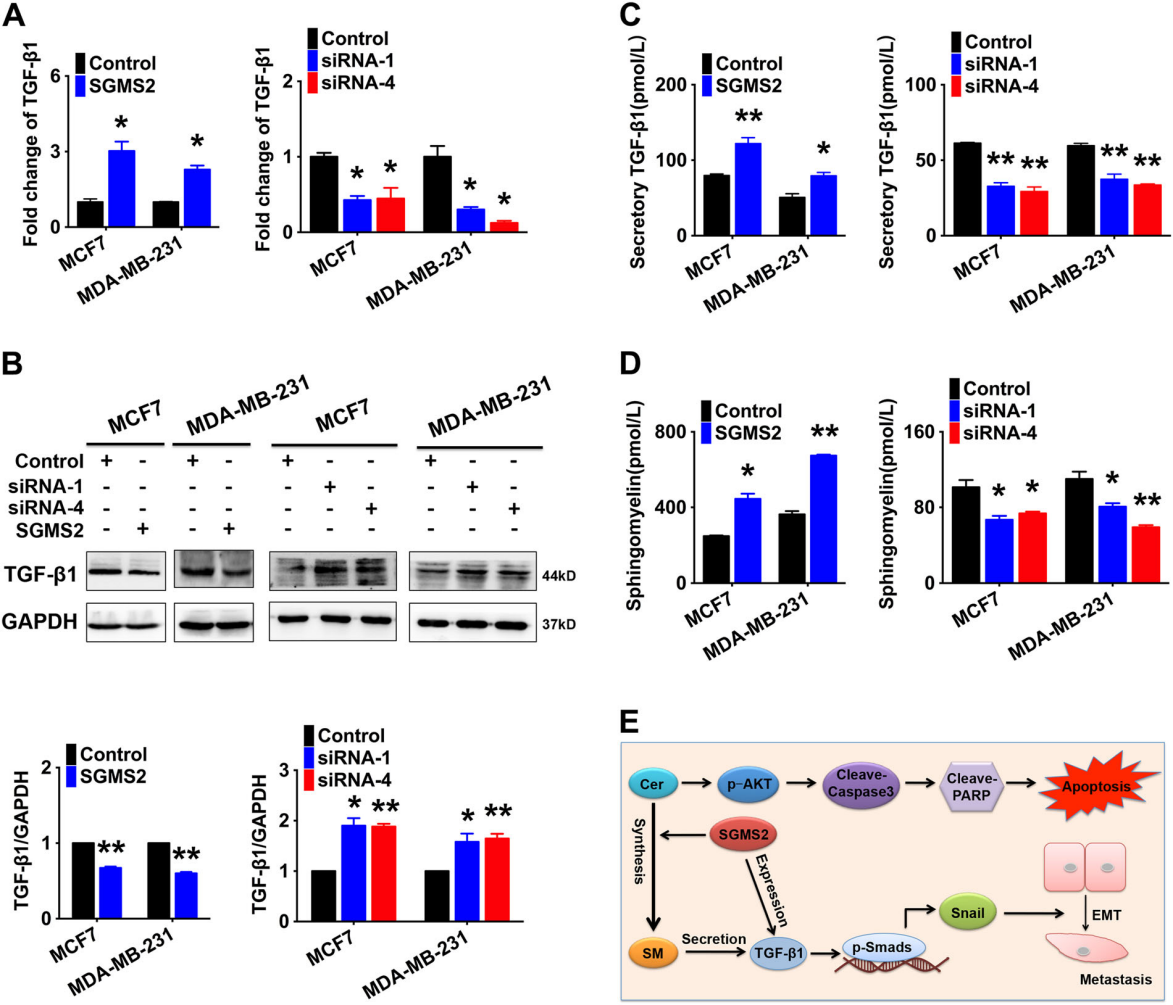
SGMS2 activates the TGF-β/Smad signalling pathway primarily by promoting TGF-β1 secretion. **a** Real-time PCR analysis of TGF-β1expression in breast cancer cells transfected with control, SGMS2 ORF constructs or anti-SGMS2 small interfering RNA oligonucleotides. Transcript levels were normalised to GAPDH expression and control groups. **b** Western blot analysis was performed to detect TGF-β1 protein levels. Representative figures are shown in the left panel. Bars in the right panel represent the expression of genes normalised to reference gene GAPDH expression and control groups. **c** ELISA assay of extracellular expression of TGF-β1 in cells treated with SGMS2 ORF construct, siRNA and their controls. **d** ELISA assay of SM expression in cells treated with SGMS2 ORF construct, siRNA and their controls. **e** Hypothetical model showing that SGMS2 promotes an aggressive breast cancer phenotype by disrupting the homoeostasis of Cer and SM. *P < 0.05 and **P < 0.01 vs. control

To determine the mechanism underlying the activation of TGF-β/Smad signalling mediated by SGMS2, we investigated the expression of TGF-β1. Real-time PCR analysis revealed that SGMS2 increased the number of TGF-β1 transcripts (Fig. 5a, Supplementary Fig. S5A), whereas immunoblotting assays showed that SGMS2 reduced the expression of intracellular TGF-β1 (Fig. 5c, Supplementary Fig. S5B). Further enzyme-linked immunosorbent assays (ELISAs) demonstrated that both transient and stable SGMS2 overexpression enhanced the secretion of TGF-β1, whereas silencing of SGMS2 reduced the expression of secretory TGF-β1 (Fig. 5b, Supplementary Fig. S5C). These findings suggest that SGMS2 significantly promotes the expression of extracellular TGF-β1 by enhancing its secretion. In addition, ELISA assays demonstrated that SGMS2 upregulation increased SM expression, while SGMS2 suppression inhibited the expression of SM (Fig. 5d, Supplementary Fig. S5D). It is well-known that SM is involved in regulation of cell secretory activity^16,17^. Thus, we supposed that SGMS2 promotes TGF-β1 secretion by upregulating the expression of SM. In conclusion, our hypothetical model showed that aberrant upregulation of SGMS2 disrupts the hemeostasis of ceramide and SM, which activates the Cer-associated apoptosis pathway and TGF-β/Smad pathway, leading to BRC development and progression (Fig. 5e).

### SGMS2 promotes the proliferation and metastasis of breast cancer in vivo

**Fig. 6:**
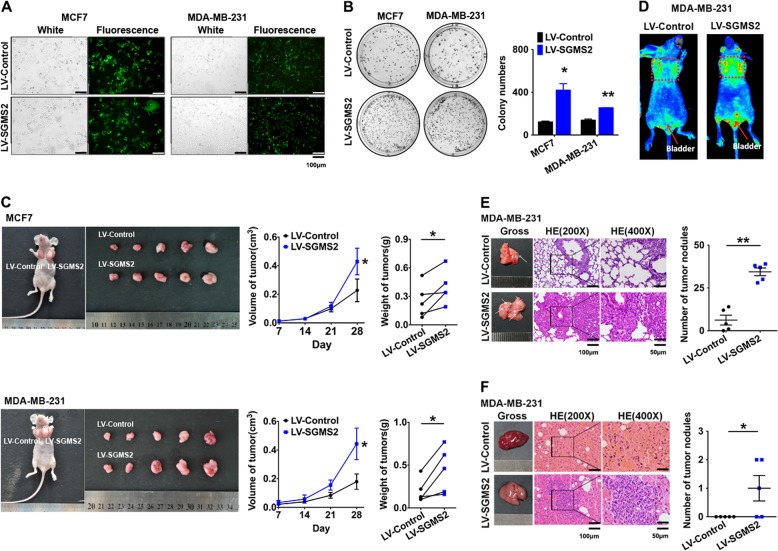
Stable overexpression of SGMS2 promotes breast cancer growth and progression in vivo. **a** Immunofluorescence assay of SGMS2 expression in breast cancer cells treated with SGMS2-lentivirus or control-lentivirus. Representative figures are shown. **b** Representative pictures (left panel) and quantification (right panel) of the colony formation assay for crystal violet-stained cells. **c** Tumour cells were injected subcutaneously into the fat pad of nude mice to determine the rate of tumourigenesis. A figure representing tumour formation is shown. Tumour volume in the backs of nude mice injected with the indicated cells was measured. The data for all primary tumours are expressed as the mean ± SD. The histogram summarises the volume of tumours derived from the indicated cells 28 days after subcutaneous implantation. **d** Caliper IVIS Lumina II (Caliper Life Sciences, Hopkinton, MA, USA) was used for optical imaging of tumour growth in lungs; representative figures are shown. **e** Tumour cells were injected into nude mice through the tail vein to evaluate their lung homing potential. The number of metastatic nodules in lungs from individual mice was determined under a microscope. The magnified areas indicate the number of metastatic nodes in the lung. **f** Tumour cells were injected into nude mice through the tail vein to evaluate their lung homing potential. The number of metastatic nodules in the livers of individual mice was determined under a microscope. The magnified areas indicate the number of metastatic nodes in the liver. *P < 0.05 and **P < 0.01 vs. Control

A lentivirus (LV)-based system (Genepharma, Shanghai, China) was used to investigate the biological function of SGMS2 *in vivo*. Immunofluorescence assays determined the LV transfection efficiency (Fig. 6a), and real-time PCR assays confirmed a significant increase in SGMS2 transcripts in LV-SGMS2-transfected cells compared with LV-control-transfected cells (*P* < 0.05, Supplementary Fig. S6A). For in vitro studies, stable overexpression of SGMS2 in MCF-7 and MDA-MB-231 cells led to an increased potential for cell proliferation and clone formation and disturbed the Cer-associated apoptosis pathway (Fig. 6b, Supplementary Figs. S6B, C, S7). In addition, stable overexpression of SGMS2 promoted migration/invasion, motility and EMT in both MCF-7 and MDA-MB-231 cells after transduction with LV (Supplementary Figs. S8–S11).

A subcutaneous tumour mouse model was used to determine the effect of SGMS2 on the growth of breast cancer cells in vivo. We found that SGMS2 significantly promoted the proliferation of tumour cells in vivo in both MCF-7 and MDA-MB-231 cell lines (*P* < 0.05, Fig. 6c). To determine the effect of SGMS2 on homing capacity in vivo, 5 × 10^6^ cancer cells were injected into nude mice through the tail vein to observe the rate of nodule formation in the lungs and liver. Compared with controls, more and bigger tumour nodules were found in the lungs of mice that received MDA-MB-231 cells overexpressing SGMS2 (*P* < 0.05, Fig. 6d, e). Interestingly, metastatic liver lesions were found in 3 mice in the MDA-MB-231/LV-SGMS2 group, while no metastatic nodules were found in mice in the MDA-MB-231/LV-control group (*P* < 0.05, Fig. 6f). However, both the MCF-7/LV-SGMS2 and MCF-7/LV-control groups showed a complete absence of tumour nodules in the lungs and liver (Supplementary Fig. S12).

## Discussion

Although many molecules are involved in the synthesis of SM from Cer^5,18^, SGMSs, especially SGMS2, remain the driving pathway that accounts for this synthesis in mammals^19,20^. Many studies have demonstrated that aberrant downregulation of Cer promotes cancer cell proliferation by inhibiting cell apoptosis^21–23^. In addition, studies have revealed that SM plays a vital role in abnormal activation of tumour-associated signalling pathways, including the mTOR^24^ and RAS-MAPK signalling pathways^25^. Studies of human cervical carcinoma^26^ and leukaemia^11,27^ have even revealed that the effect of SGMS2 on cell proliferation and differentiation involves the homoeostasis of Cer and SM. Although the role of SGMS2 in the development and progression of breast cancer is still unknown, the findings mentioned above strongly suggest that SGMS2 may act as a tumour promoter in breast cancer.

A previous study revealed that high-SGMS activity is positively associated with the incidence of haematological malignancies^28^. However, our study found no significant difference in SGMS2 expression between breast cancer tissue and paired normal tissue, which suggests that aberrant expression of SGMS2, is unrelated to the incidence of breast cancer. However, it was intriguing that the expression of SGMS2 was higher in patients with lymph node metastasis than in those without metastasis, which strongly suggests that SGMS2 is associated with tumour metastasis in breast cancer patients. However, the role of SGMS2 in tumour metastasis and its underlying mechanism are still not clear.

To further elucidate the exact role of SGMS2 in breast cancer, we carried out gain- and loss-of-function studies in vitro and in vivo. SGMS2 expression was positively associated with cell viability and colony formation and negatively associated with cell apoptosis through its inhibition of the Cer-related pathway, which was consistent with the results of studies in lymphocytes, hepatocytes, astrocytes and fibroblasts^29–31^. In addition, our studies revealed that SGMS2 significantly promoted in vitro migration, motility and invasiveness of breast cancer cells through EMT. Notably, a significant change in mesenchymal type was observed only in MCF-7 cells after transient and stable SGMS2 overexpression. However, a significant change in epithelial type was observed only in MDA-MB-231 cells with decreased SGMS2 expression. Moreover, tumour-lung homing was only found in subjects receiving the MDA-MB-231 cell line, while subjects receiving the MCF-7 cell line showed a complete absence of nodule formation in lungs. We considered that these different results between the MCF-7 and MDA-MB-231 cell lines may be attributed to their distinct characteristics and gene-expression profiles^10,32–34^.

The classic TGF-β/Smad signalling pathway is well-known to play a crucial role in EMT initiation by regulating downstream expression of proteins, such as Snail/Slug, ZEB1/2 and Twist family proteins^35–37^. Our studies revealed that SGMS2 indeed activates the TGF-β/Smad signalling pathway and subsequently increases the expression of its downstream protein Snail. In addition, we found that aggressive breast cancer cell phenotypes were recovered when the TGF-β/Smad signalling pathway was specifically arrested in SGMS2 overexpression cells by pirfenidone, and the expression of EMT-related markers was also reversed. These findings strongly suggest that SGMS2 promotes an aggressive breast cancer cell phenotype by activating the TGF-β/Smad signalling pathway.

It is well-known that TGF-β1 plays an important role in activating the TGF-β/Smad signalling pathway^38,39^. To determine the mechanism underlying the activation of TGF-β/Smad signalling pathway mediated by SGMS2, we investigated the expression and secretion of TGF-β1. Our studies found that SGMS2 indeed increased the transcription and secretion of TGF-β1. However, we found significantly decreased expression of intracellular TGF-β1 with transient SGMS2 overexpression. Some studies have revealed the potential role of SGMSs in cellular secretory activity^40^. SGMSs can promote the transport of vesicular stomatitis virus G protein and enhance the secretion of insulin in rat β cells^41^. A recent study even determined that SGMSs regulate the defensive activity of neutrophils by promoting the release of an antifungal factor^42^.These findings strongly suggest that SGMS2 promotes the expression of extracellular TGF-β1 primarily by enhancing its secretion level.

SM is a dominant component of membranous vesicles, including early/late and recycling endosomes and lysosomes^43^. Studies have confirmed that SM is broadly required for cell secretory activity^40^, thus suppression of SM significantly inhibits the secretion of influenza haemagglutinin^44^. A recent study also revealed that a short form of the auxiliary TGF-β receptor endoglin selectively interacts with SM and is released into circulation via SM-mediated exosomes^45^. Considering the effects of SGMS2 on cell secretory activity and SM homoeostasis, we propose that SGMS2 enhances the secretion of TGF-β1 by promoting the expression of SM.

## Conclusion

In summary, we found increased expression of SGMS2 in patients with metastatic breast cancer. The aberrant expression of SGMS2 disrupted the hemeostasis of Cer and SM. In turn, suppression of Cer expression led to enhanced cell proliferation potential through inhibition of cell apoptosis. SGMS2 increased the expression of TGF-β1 by upregulating SM, which subsequently activated the TGF-β/Smad signalling pathway and promoted EMT in breast cancer cells, thus increasing the migration and invasiveness of breast cancer cells. These findings may help confirm the mechanisms underlying breast cancer development and progression and allow us to devise novel anticancer therapies.

## Supplementary information

Supplementary results

Supplementary materials and method

Supplementary table

## Data Availability

All data and materials would be available if required.
