# Supplementary material for: Sphingomyelin synthase 2 promotes an aggressive breast cancer phenotype by disrupting the homoeostasis of ceramide and sphingomyelin
Source: Cell Death Dis. 2019 Feb 15;10(3):157. doi: 10.1038/s41419-019-1303-0 (PMC6377618; doi:10.1038/s41419-019-1303-0)
Supplement: Supplementary file 1 — Supplementary results [file 41419_2019_1303_MOESM1_ESM.pdf]

Supplementary Figures

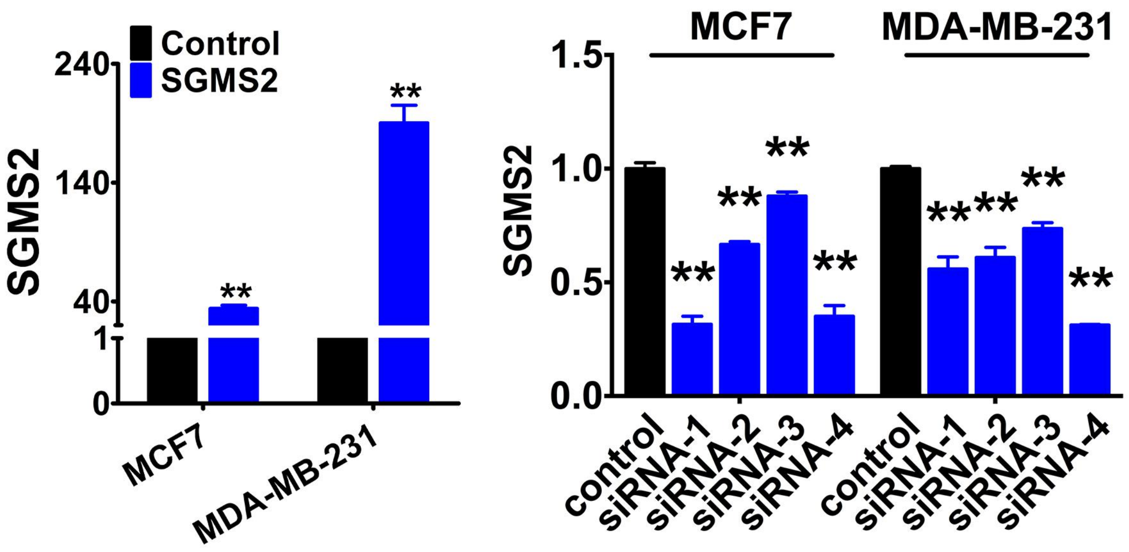

Figure S1, related to Figure 1. The efficiency of SGMS2 ORF constructs and anti-SGMS2 small interfering RNA oligonucleotides (siRNA) on MCF-7 and MDA-MB-231 cells. Real-time PCR assays were performed here. All samples were normalized to internal controls GAPDH, and fold changes were calculated through relative quantification (2-ΔΔCT).

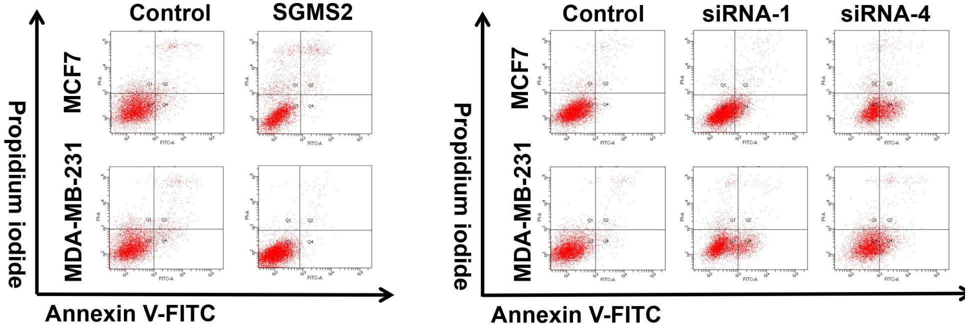

Figure S2, related to Figure 1. The regulation of SGMS2 on cell proliferation is related with cell apoptosis. Annexin V staining showed the percentage of cells undergoing apoptosis in MCF7 and MDA-MB-231 cells treated with SGMS2 ORF constructs, siRNA and their control.

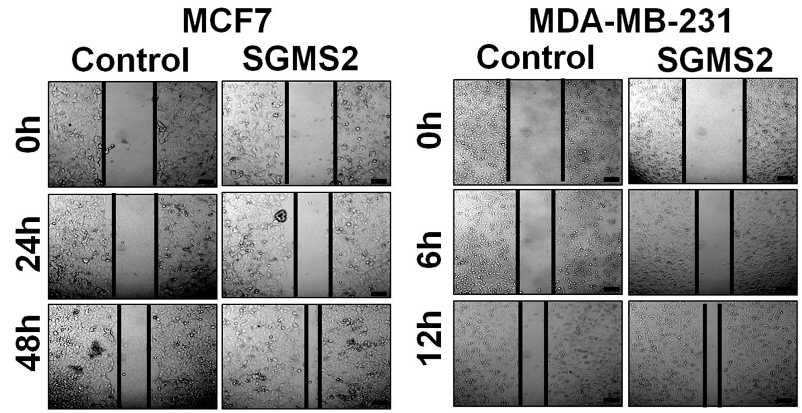

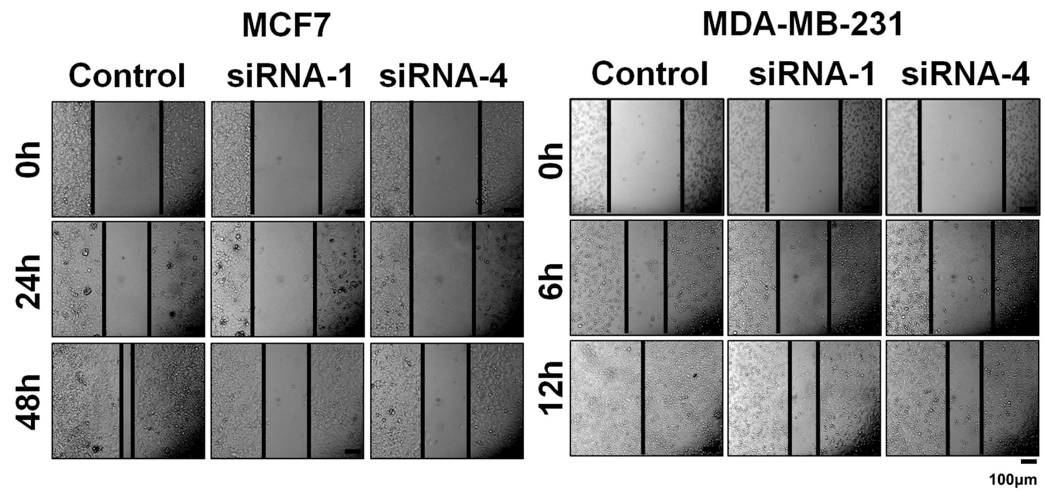

Figure S3, related to Figure 2. The representative figures of wound healing assay for MCF7 and MDA-MB-231 cells that were treated with SGMS2 ORF constructs, siRNA and their control. The wounds were measured under a microscope in three randomly selected fields.

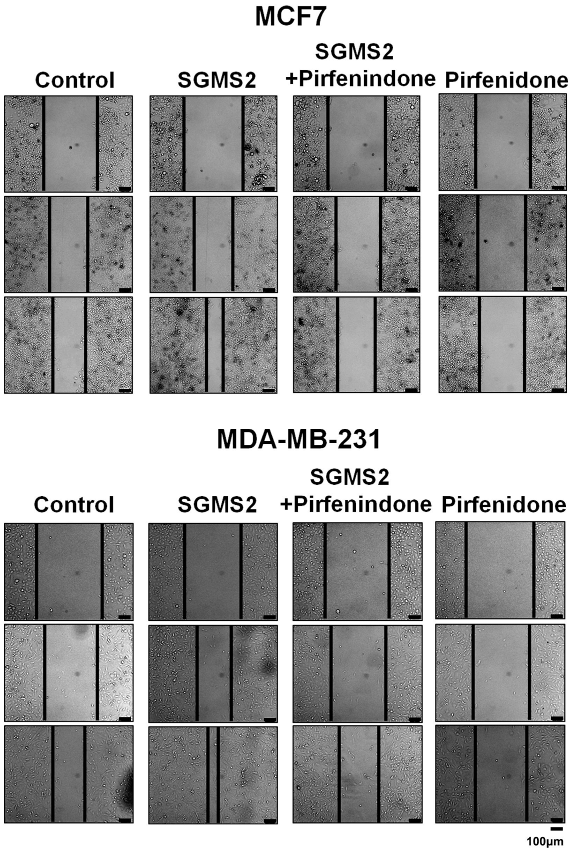

Figure S4, related to Figure 4. The representative figures of wound healing assay for MCF7 and MDA-MB-231 cells that were treated with SGMS2 ORF constructs, their control and/or Pirfenidone. The wounds were measured under a microscope in three randomly selected fields.

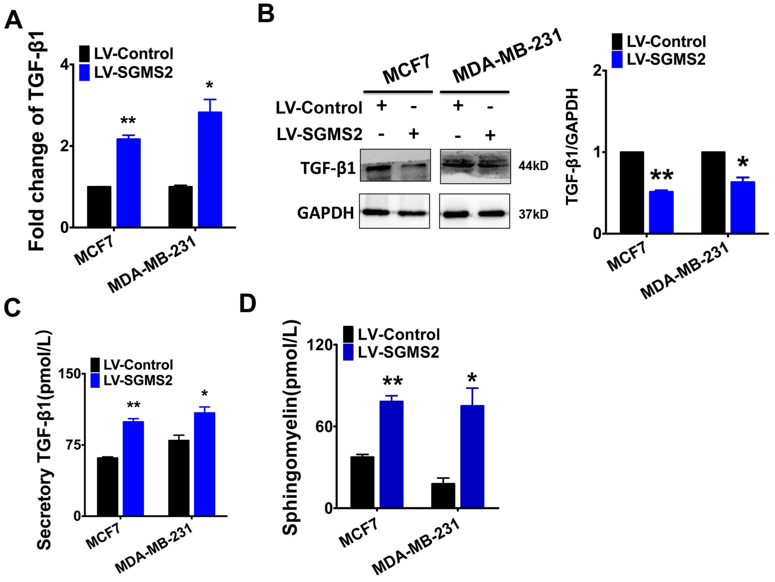

Figure S5, related to Figure 5. Stable over-expression of SGMS2 promotes the secretion of TGF-β1. (A) Real-time PCR analysis of TGF-β1 expression in cells treated with SGMS2-lentivirus and, control-lentivirus. (B) Western blot analysis was performed to detect TGF-β1. Representative figures are shown in the left panel. Bars in the right panel represent the expression of genes normalized to reference gene GAPDH expression and control groups. (C) ELISA assay of Secretory TGF-β1 in SGMS2-transduced cells. (D) Stable over-expression of SGMS2 promotes the expression of Sphingomyelin. ELISA assay of sphingomyelin in SGMS2-lentivirus and , control- lentivirus. $^{*}P<0.05$, $^{**}P<0.01$, vs LV-Control.

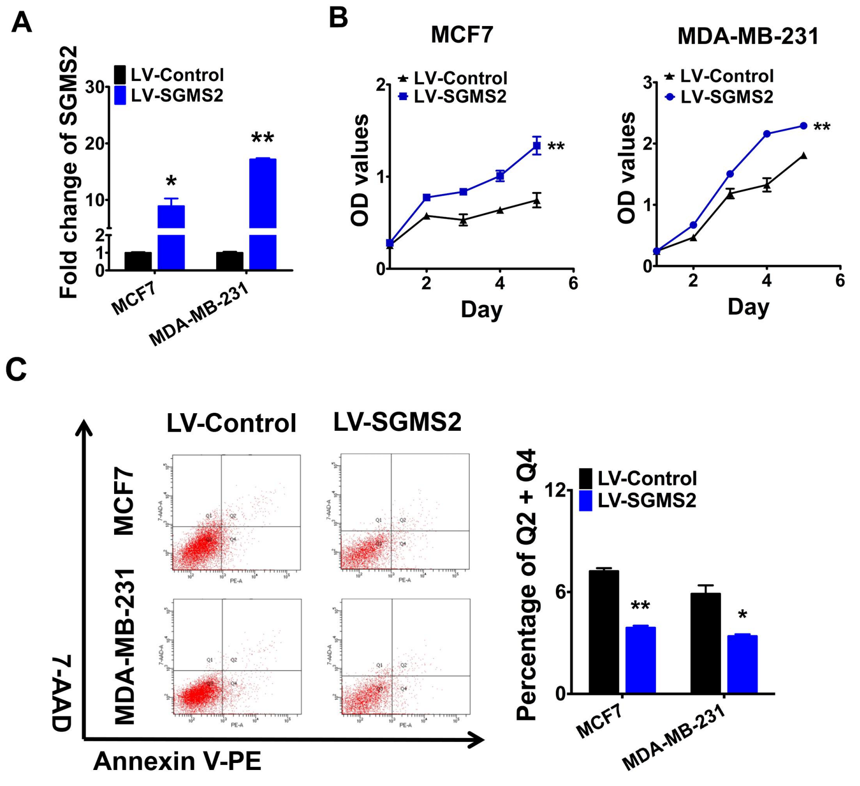

Figure S6, related to Figure 6. Stable over-expression of SGMS2 promotes cells proliferation by inhibiting cell apoptosis. (A) The efficiency of SGMS2 lentivirus on MCF-7 and MDA-MB-231 cells was evaluated by real-time PCR assays. (B) The effects of stable over-expression of SGMS2 on cell proliferation was evaluated by CCK-8 assay. (C) Representative figures for genes Annexin V staining analysis are shown (left panel). Bars in the right panel represent the percentage of cells undergoing apoptosis in MCF7 and MDA-MB-231 cells transfected with control-lentivirus and SGMS2-lentivirus. $^{*}P<0.05$, $^{**}P<0.01$, vs LV-Control.

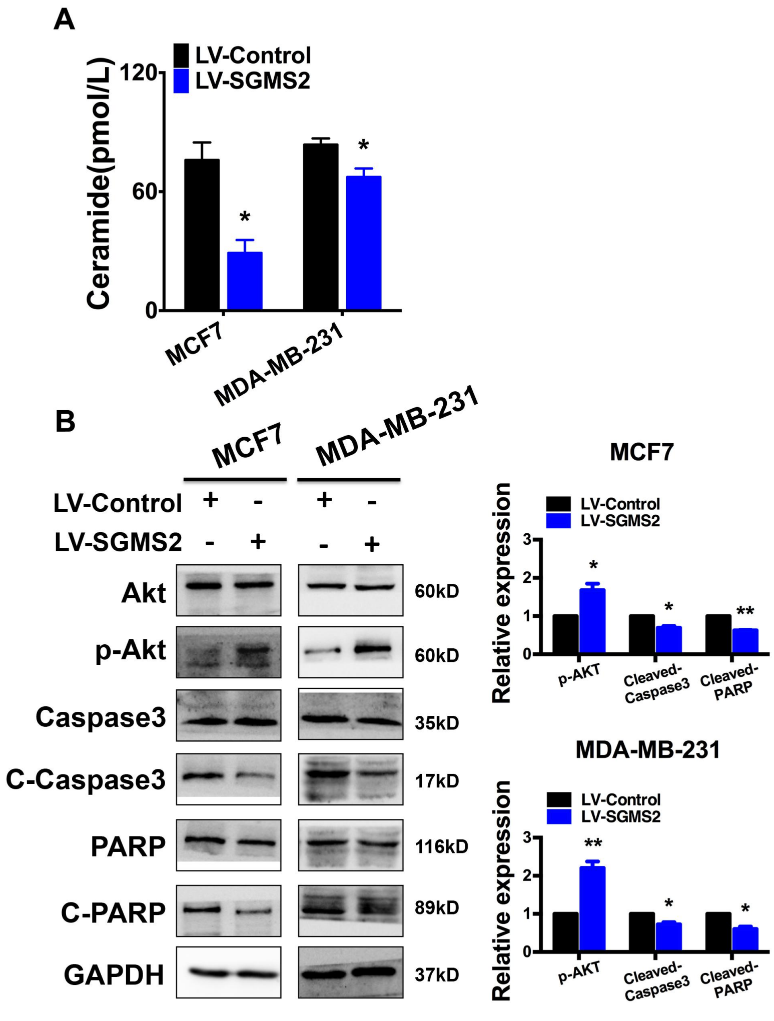

Figure S7, related to Figure 6. Stable over-expression of SGMS2 reduces breast cancer cell apoptosis. (A) ELISA assay of ceramide in cells treated with SGMS2-lentivirus and , control-lentivirus. (B) Western blot analysis was performed to detect cell apoptosis signaling pathway-associated proteins. Representative figures are shown in the left panel. Bars in the right panel represent the expression of genes normalized to reference gene Akt, Caspase3, or PARP expression and control groups. “C-” indicated “Cleave-”, $^{*}P<0.05$, $^{**}P<0.01$, vs LV-Control.

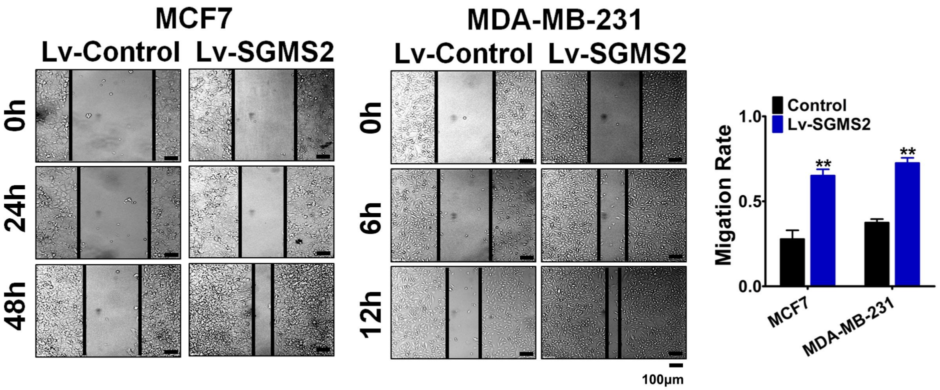

Figure S8, related to Figure 6. Stable over-expression of SGMS2 promotes breast cancer cell migration. The representative figures of wound-healing assay of breast cancer cells treated with SGMS2-lentivirus, and control-lentivirus. The cells were counted under a microscope in five randomly selected fields. Bars in the right panel represent the migration rate of cells. $^{**}P<0.01$, vs LV-Control.

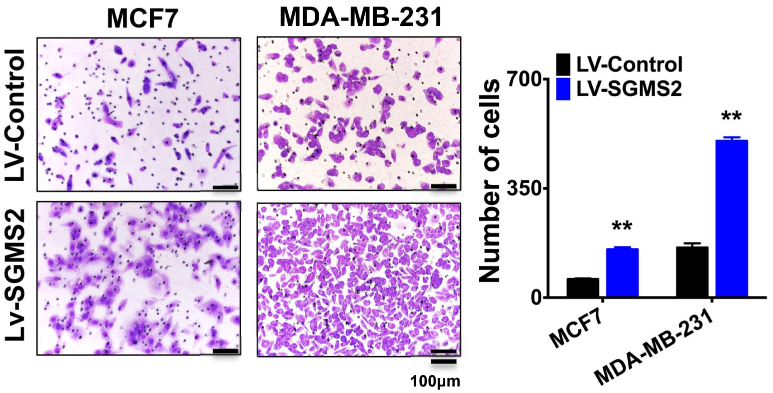

Figure S9, related to Figure 6. Stable over-expression of SGMS2 promotes breast cancer cell motility. The representative figures of transwell assay of breast cancer cells treated with SGMS2-lentivirus, and control-lentivirus. The cells were counted under a microscope in five randomly selected fields. Bars in the right panel represent the number of invaded cells. $^{**}P < 0.01$, vs LV-Control.

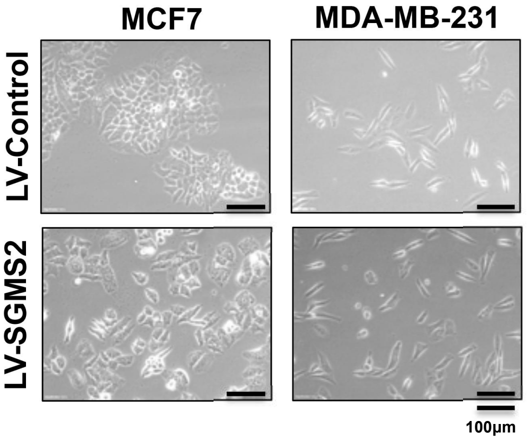

Figure S10. related to figure 6. Stable over-expression of SGMS2 promotes EMT of breast cancer cell. The representative figures of cell morphology assay of breast cancer cells treated with SGMS2-lentivirus, and control-lentivirus.

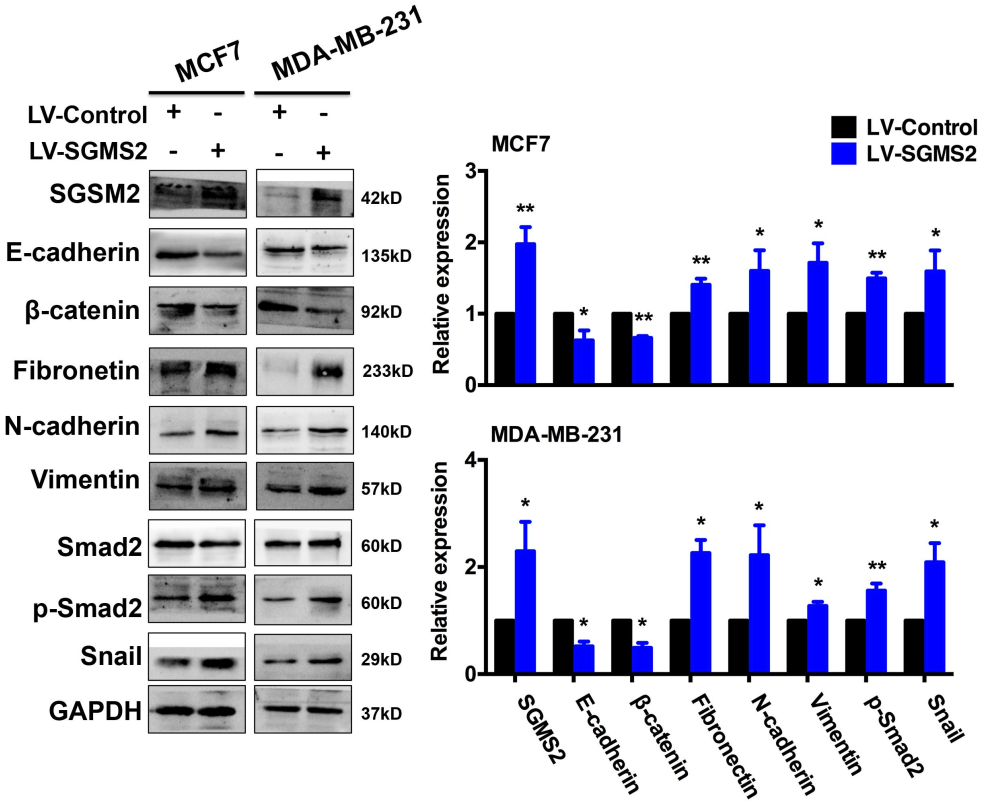

Figure S11, related to Figure 6. Stable over-expression of SGMS2 promotes TGF-β/Smad signaling pathway and EMT of breast cancer cell. Western blot analysis was performed to detect EMT and TGF-β/Smad signaling pathway-associated proteins. Representative figures are shown in the left panel. Bars in the right panel represent the expression of genes normalized to reference gene GAPDH or Smad2 expression and control groups.

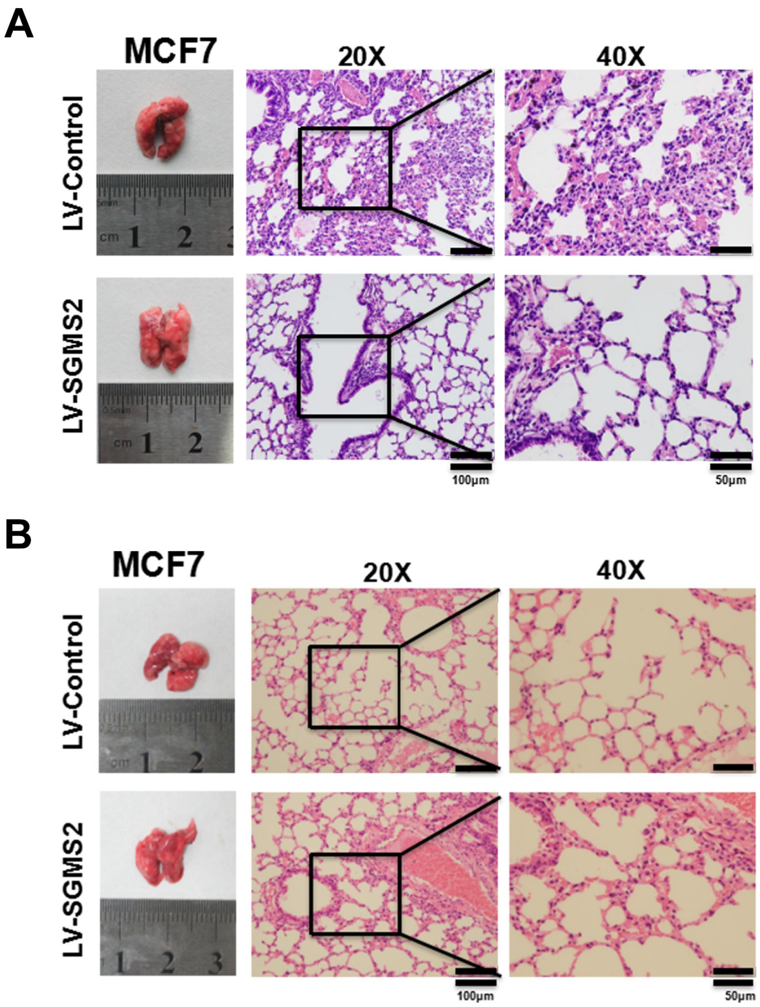

Figure S12. related to figure 6. Stable over-expression of SGMS2 play no role in homing capacity of MCF7 cell lines *in vivo*. (A) 5×10^6 tumor cells were injected into nude mice through the tail vein to evaluate their lung homing capacity. Mice were treated with 17-estradiol as described above. (B)Same experiment was repeated on nude mice not given the treatment of 17-estradiol. The number of metastasis nodules in the lungs of individual mice was counted under a microscope.
