# Supplementary material for: Sphingomyelin synthase 2 promotes an aggressive breast cancer phenotype by disrupting the homoeostasis of ceramide and sphingomyelin
Source: Cell Death Dis. 2019 Feb 15;10(3):157. doi: 10.1038/s41419-019-1303-0 (PMC6377618; doi:10.1038/s41419-019-1303-0)
Supplement: Supplementary file 2 — Supplementary materials and method [file 41419_2019_1303_MOESM2_ESM.docx]

**Supplementary materials and methods**

**Cell proliferation assays**

Cell proliferation assays were carried out using a Cell Counting Kit 8 (CCK-8; Dojindo; Kumamoto, Japan). Cells were plated in 96-well plates at a density of 1 ×104cells per well and were cultured in the growth medium. At the indicated time points, the number of cells in triplicate wells was measured at an absorbance at 450 nm of reduced WST-8(2-(2-methoxy-4-nitrophenyl)-3-(4-nitrophenyl)-5-(2,4-disulfo-

phenyl)-2H-tetrazolium monosodium salt). Two-way ANOVA followed by multiple comparison tests were carried out for comparisons between two groups.

**Cell apoptosis analysis**

Cells (5×105) were washed with ice-cold PBS then resuspended in 500 μl of binding buffer (KeyGen Biotechnology, Nanjing, China). Annexin V-FITC (5μl)(KeyGen) and propidium iodide (5 μl) were added to the cell suspension. The mixture was then incubated at room temperature for 10 min in the dark. Analysis of apoptosis was carried out using flow cytometry.

**Cell motility analysis**

Cell from the serum-free medium (1×105 cells/100 μl) were added to the top chamber of each 8-mmpore Transwell chamber (Corning Star, Cambridge, Massachusetts). The bottom chamber was prepared using 10% FBS as a chemoattractant. Cells were allowed to migrate through the porous membrane for 48 h at 37℃. The cells that stuck to the lower surface of the membrane were treated with a fixation/staining solution (0.1% crystal violet, 1% formalin, and 20% ethanol) for visualization. The cells were counted under a microscope( in 5 randomly selected fields; original magnification:×200). At least four chambers from three different experiments were analyzed.

**Tumor invasion analysis**

Cell from the serum-free medium (1×105 cells/100 μl) were added to the top chamber of each 8-μm-pore transwell chamber (Corning Star, Cambridge, Massachusetts) with matrigel. The bottom chamber was prepared using 20% FBS as a chemoattractant. Cells were allowed to migrate through the porous membrane for 24 h - 48 h at 37℃. The cells that stuck to the lower surface of the membrane were treated with a fixation/staining solution (0.1% crystal violet, 1% formalin, and 20% ethanol) for visualization. The cells were counted under a microscope( in 5 randomly selected fields; original magnification:×200). At least four chambers from three different experiments were analyzed.

**Wound healing assay**

Confluent monolayers of cells were maintained in serum-containing growth medium for at least 6 days then maintained in serum-free medium for 24 h. A 1000-μl plastic pipette tip was used to scratch the monolayers. The wounded cells were then cultured in serum-free medium for an additional 12 h - 48 h and photographed under an inverted phase contrast microscope. Three different points were marked on the plate, and the distance between each point and the edge of the scratch wound was measured before and after cell migration. The mean migration distance (μm) was calculated by subtracting the length after 12h - 48 h from that at 0 h. The result was expressed as a migration index, i.e., the distance migrated by treated cells compared with the distance migrated by control cells. Experiments were carried out in triplicate and repeated at least five times.

**Tumor growth assay**

Four-to-five-week-old male BALB/c nude mice were purchased from the Laboratory Animal Services Centre at the Southern Medical University. Animal handling and experimental procedures were approved by the Animal Experimental Ethics Committee of Southern Medical University. For the tumor growth assay, 1×107 SGMS2 overexpression cells were injected subcutaneously into the right fat pad of each nude mice, while the control cells were correspondingly injected into the left side (*N*=5/group). To facilitate estrogen-dependent MCF-7 xenograft establishment, a pellet containing 17-estradiol was implanted subcutaneously (0.72 mg/ pellet, 60 day release; Innovative Research of America^[1](#_ENREF_1)^). The tumor volume was calculated using the following formula: V=0.5×D×d^2^, where V represents volume, D represents the longitudinal diameter, and d represents the latitudinal diameter.

**Tumor metastasis assay**

To determine the lung homing potential of cancer cells *in vivo*, we injected 5×106cells infected with LV-SGMS2 and LV-control into nude mice (*N*=5/group) through the tail vein. Caliper IVIS Lumina II (Caliper Life Sciences, Hopkinton, MA, USA) was used for optical imaging of tumor growth and the formation of metastasis lesions. The mice were all sacrificed 6 weeks later, at which time individual organs were removed and metastatic tissue was analyzed using hematoxylin and eosin stains.

1 Xu, Y. *et al.* Effect of estrogen sulfation by SULT1E1 and PAPSS on the development of estrogen-dependent cancers. *Cancer science* **103**, 1000-1009, doi:10.1111/j.1349-7006.2012.02258.x (2012).
