# Supplementary material for: Sphingomyelin synthase 2 promotes an aggressive breast cancer phenotype by disrupting the homoeostasis of ceramide and sphingomyelin
Source: Cell Death Dis. 2019 Feb 15;10(3):157. doi: 10.1038/s41419-019-1303-0 (PMC6377618; doi:10.1038/s41419-019-1303-0)
Supplement: Supplementary file 3 — Supplementary table [file 41419_2019_1303_MOESM3_ESM.docx]

**Supplementary table S1.** RT-PCR primer sequences for human genes

| **Gene** | **Forward primer** | **Reverse primer** | **Product length** |
| --- | --- | --- | --- |
| **TGF-β1** | **CGCTGCCCATCGTGTACTA** | **ACACAGAGATCCGCAGTCCT** | **240bp** |
| **SGMS2** | **CTTAGCCCTCCACTCCC** | **CAGAATCTGCGTCCCAC** | **216bp** |
| **RPLP0** | **AGCCCAGAACACTGGTCTC** | **ACTCAGGATTTCAATGGTGCC** | **97bp** |
| **GAPDH** | **GGAGCGAGATCCCTCCAAAAT** | **GGCTGTTGTCATACTTCTCATGG** | **197bp** |

| **Gene** | **Sense** | **Antisense** |
| --- | --- | --- |
| **siRNA-1** | **CGAUUAGAAAGAUGAACAATT** | **UUGUUCAUCUUUCUAAUCGTT** |
| **siRNA-2** | **GGUUCAGAAGAUUGGUGAATT** | **UUCACCAAUCUUCUGAACCTT** |
| **siRNA-3** | **CGCUGUAACCAAAGGUAUATT** | **UAUACCUUUGGUUACAGCGTT** |
| **siRNA-4** | **CGAACACUACACUAUCGAUTT** | **AUCGAUAGUGUAGUGUUCGTT** |

**Supplementary table S2.**siRNA sequences for SGMS2
